# Electrical storm after percutaneous coronary intervention: Ischemia, reperfusion injury, or channelopathy?

**DOI:** 10.1002/joa3.12382

**Published:** 2020-06-12

**Authors:** Carolina Vila Chã Vaz Saleiro, Joana Maria da Silva Barbosa Guimarães Ribeiro, Rogério Paiva Cardoso Teixeira, João Pedro Gameiro Lopes, Diana Catarina Ferreira de Campos, Carolina Maria Negrier Lourenço, Lino Manuel Martins Gonçalves

**Affiliations:** ^1^ Department of Cardiology Centro Hospitalar e Universitário de Coimbra Coimbra Portugal; ^2^ Faculdade de Medicina da Universidade de Coimbra Coimbra Portugal

**Keywords:** Brugada syndrome, coronary artery disease, electrical storm, percutaneous coronary intervention

1

Institution of timely treatment for electrical storm is crucial but challenging. Beta‐blockers and amiodarone are often first line as they are effective in suppressing ventricular arrythmias (VAs) and have a favorable safety profile. However, in cases of Brugada Syndrome (BrS) and early‐repolarization syndromes, these drugs will be largely ineffective, and pathology‐guided treatment is required. We present a case that highlights the importance of early recognition of the precipitating mechanisms of electrical storm.

A 66‐year‐old man was referred for percutaneous intervention (PCI) due to stable angina with positive functional imaging test. Previous medical history included hypertension, dyslipidemia, and overweight and he was medicated accordingly. There was no family history of cardiac disease or sudden death. Angiography showed three‐vessel coronary artery disease: the left anterior descending artery (LAD) had a critical distal lesion, which was treated with PCI, and an intermediate ostial lesion that was deferred; the circumflex artery (LCx) was diffusely diseased; and the right coronary artery (RCA) had two significant lesions—90% proximal and 75% distal stenosis that were treated with PCI. The procedure was successful and uneventful.

The patient was admitted to the cardiology ward for surveillance. Ten hours later, he reported mild atypical chest pain. Electrocardiogram (ECG) showed de novo saddleback ST‐segment elevation from V1 to V3 consistent with a type‐2 Brugada pattern (BrP). The heart rate ranged between 50 and 60 beats/minute, similar to ambulatory heart rate. Twenty hours post‐PCI, the patient developed multiple episodes of polymorphic ventricular tachycardia and fibrillation requiring defibrillation (six shocks in total, over a period of 3 hours).

No fever or ionic disturbances were documented. Echocardiography showed good biventricular function, with regional wall motion abnormalities (LCx and RCA territories). The ECG after the second shock revealed a type‐1 BrP.

Two primary causes for the VAs were considered: acute ischemia and BrS. Initially, ischemia was thought to be more likely and esmolol and amiodarone were administered. As this approach was ineffective, treatment was switched to isoproterenol (1 ug/min perfusion) and overdriving ventricular pacing at the right ventricle, at 80 beats/minute, as BrS was presumed to be more likely. The last episode of VA occurred a few minutes after isoproterenol was initiated. Emergent coronary angiography excluded acute ischemia. VAs were successfully suppressed by these measures. Three days later, angioplasty of the ostial LAD was performed and the temporary pacemaker was removed due to sustained electrical stability. Isoproterenol was progressively withdrawn, and oral quinidine 200 mg four times a day was initiated (progressively tapered to a final dose of 200 mg twice daily which was maintained at hospital discharge).

Magnetic resonance showed subendocardial late gadolinium enhancement in the lateral and posterior walls (42 mm), consistent with ischemic cardiomyopathy. The patient received a cardioverter defibrillator and was discharged home 12 days after the initial PCI.

Genetic screening for the most common mutations associated with BrS (CACNA1C, CACNB2b, SCN10A, and SCN5A genes) was negative. After 1‐year follow‐up, the patient remains free of cardiovascular events, under oral quinidine. No significant arrhythmias were detected on routine ICD evaluations and resting ECGs shows no BrP.

BrS has been associated with mutations in inward Na^+^ (*I*_Na_) and L‐type Ca^2+^ channels that decrease phase‐0 rapid depolarization and mutations in the transient outward K^+^ (*I*_to_) channel that increase phase‐1 rapid repolarization.^1^ Although the mechanisms are incompletely understood, functional re‐entry is commonly accepted as the basis for arrhythmogenesis.^1^ *I*_to_ expression is greater in the right ventricle (RV) outflow tract and decreases from the epicardium to the endocardium, generating a transmural gradient that, in pathological conditions such as decreased phase‐0 rapid repolarization or gain‐of‐function of *I_to_* channels, provides a substrate for reentrant arrhythmias.^1^

Because of the particularities of its treatment, timely diagnosis of BrS is mandatory. Once this diagnosis was assumed, proper treatment was initiated with a favorable response. The efficacy of isoprosterenol lies in the fact that it augments L‐type calcium channels, preventing loss of action potential dome. Overdrive pacing may play a role in the termination of VAs, by inducing an extrastimuli and stopping the reentrant circuit. Moreover, *I*_to_ currents are less prominent at faster heart rates; thus, by increasing the basal heart rate, rapid pacing may have a role in decreasing the risk of arrhythmia generation. Quinidine seems to have good long‐term effectiveness in preventing VAs due to its *I*_to_ blocking properties.

We also questioned the underlying trigger of the VAs. While Brugada phenocopies have been reported during RCA angiography and similarities between the generation of ischemic ST‐segment elevation and BrP have been proposed.^2^ This is, to the best of our knowledge, the first report of a de novo BrP unmasked after PCI associated with the clinical manifestation of VAs. Acute ischemia of the conus branch can induce BrP and precipitate VAs.^3^ However, the fact the clinical manifestations developed several hours after intervention and the coronary angiogram remained unchanged weight against the theory that acute ischemia was the trigger.

The mechanisms by which the ST‐segment changes vary over time and the arrhythmias that are triggered are also poorly understood. In this case, there seems to be a direct myocardial insult. Moreover, the patient reported mild chest pain after PCI of a proximal RCA lesion, suggesting reperfusion injury and justifying the involvement of the RV. Swelling‐activated chloride channels (*I*_Cl,swell_) have been reported to be activated during ischemia/reperfusion, generating an outward Cl^−^, that has been suggested to decrease phase‐0 inward sodium currents (*I*_Na_).^4^ We hypothesize that in this case, the activation of *I*_Cl,swell_ (further) decreased phase‐0 *I*_Na_, triggering the VAs. It remains unclear, however, whether ischemia–reperfusion injury would be enough to justify this clinical presentation, particularly outside the context of an acute coronary syndrome, or if a pre‐existing channelopathy could have been exacerbated after PCI. Interestingly, a recent case series suggested that CAD‐related but non‐ischemia‐driven drug‐refractory VAs may have a similar background to BrS (and other arrhythmogenic syndromes with a structurally normal heart) and display a remarkable response to quinidine, although in this study there is no mention of a BrP preceding the VAs.^5^

This case highlights the importance of early recognition of the precipitating mechanisms of electrical storm and raises the possibility that reperfusion injury may precipitate VAs in patients with BrS.

### CONFLICT OF INTEREST

The authors have no conflict of interest to declare.

REFERENCES1

Meregalli
PG
, 
Wilde
AA
, 
Tan
HL
. Pathophysiological mechanisms of Brugada syndrome: depolarization disorder, repolarization disorder, or more?
Cardiovasc Res. 2005;67(3):367–78.1591357910.1016/j.cardiores.2005.03.0052

Bowers
RW
, 
O’Kane
P
, 
Balasubramaniam
RN
. Type 1 Brugada ECG unmasked by intracoronary contrast media. Heart. 2013;99:147–8.2291785010.1136/heartjnl-2012-3024653

Ogano
M
, 
Iwasaki
Y‐K
, 
Morita
N
, 
Tanabe
J
, 
Shiiba
K
, 
Miyauchi
Y
, et al. Proarrhythmic ECG deterioration caused by myocardial ischemia of the conus branch artery in patients with a brugada ECG. Pattern. Pacing Clin Electrophysiol. 2010;34(3):e26–e29.10.1111/j.1540-8159.2010.02742.x203534164

Baumgarten
CM
, 
Browe
DM
, 
Ren
Z
. Swelling‐ and stretch‐activated chloride channels in the heart: regulation and function In: KamkinA, KiselevaI, editors. Mechanosensitivity in cells and tissues. Moscow: Academia; 2005.212907645

Viskin
S
, 
Chorin
E
, 
Viskin
D
, 
Hochstadt
A
, 
Halkin
A
, 
Tovia‐Brodie
O
, et al. Quinidine‐responsive polymorphic ventricular tachycardia in patients with coronary heart disease. Circulation. 2019;139(20):2304–14.3069626710.1161/CIRCULATIONAHA.118.038036
